# Investigation of bovine abortion and stillbirth/perinatal mortality - similar diagnostic challenges, different approaches

**DOI:** 10.1186/s13620-020-00172-0

**Published:** 2020-09-04

**Authors:** John F. Mee

**Affiliations:** Animal and Bioscience Research Department, Teagasc, Moorepark Research Centre, Fermoy, Co. Cork Ireland

**Keywords:** Cattle, Abortion, Stillbirth, Perinatal mortality, Investigation, Necropsy, Cause-of-death, Ultimate, Proximate, Time-of-death

## Abstract

This pracademic paper reviews current bovine foetopathy (abortion and stillbirth) case definitions, reporting and triage, and causes and time-of-death and proposes veterinary practitioner-focused investigative standard operating procedures (SOPs). Issues of under- and over-triage and intra-institutional SOP harmonisation are also discussed. It is proposed that an ‘observable abortion’ (120–260 days of gestation) is a more practitioner-friendly definition of abortion for reporting and benchmarking purposes and that the term ‘peristillbirth’ can replace stillbirth and perinatal mortality. Diagnosis of bovine foetopathy involves an investigative triad of the farmer, veterinary practitioner and the veterinary diagnostic laboratory. However, the poor sensitivity of abortion reporting undermines the value of currently adopted scanning/passive surveillance; parallel active surveillance/sentinel herd models should also be employed. The approach to abortion investigation differs from that of peristillbirth. The former should include collecting a herd and case history, examination and sampling of dam and cohorts and sampling of the foetus and placenta. A sample selection decision tree is provided to assist test selection. In peristillbirths, non-infectious and periparturient causes-of-death are more important hence the anamnesis must focus on peristillbirth risk factors and calving management. The foetopsy, while including the sampling menu appropriate to aborted foetuses, must also include a detailed internal and external examination of the carcass for lesions indicative of periparturient causes-of-death. In addition, for aborted foetuses the time-of-death is not important as the foetus is generally not viable; however, for the peristillbirth the time-of-death is critical as it provides useful information for the farmer to address modifiable risk factors and to alter their perinatal management. Reporting of the ultimate cause-of-death is more useful to prevent future abortions and peristillbirths though the proximate cause-of-death is often reported in the absence of a complete clinical anamnesis. Finally, the common reasons for diagnosis not reached (DNR) and the limitations of current investigative approaches are discussed.

## Background

Reported bovine abortion rates vary between approximately 0.5 and 10% [1]. Perinatal calf mortality (0-48 h) rates vary between approximately 2 [2] and 10% [3], internationally. These losses represent economic, welfare and societal concerns [4]. Abortion (20 to 50% [5];) and stillbirth (30 to 75% [6];), diagnosis rates do not appear to have improved over time internationally despite the development of molecular diagnostic techniques and the discovery of new foetopathogens, e.g. Schmallenberg virus [7]. While reasons for the overall low diagnostic rates from foetal investigations are detailed hereunder, this paper addresses this challenging conundrum with a view to veterinary practitioner-led improvements in diagnostic rates. The differing proportions of infectious and non-infectious and pre- and periparturient aetiologies of abortions and stillbirths are emphasised, hence though the diagnostic challenge is similar, the approaches to diagnosis differ.

The investigative triad involving the farmer, veterinary practitioner and veterinary pathologist is critical to better investigative outcomes [8]. From the farmer’s perspective, under and late reporting and placental non-submission are significant issues which can be improved. Veterinary practitioners can be more pro-active in awareness raising and client education regarding index case reporting, investigative thresholds, pro forma anamnesis recording and advising clients of possible causes of foetal loss. In addition, with closures of many government veterinary diagnostic laboratories, veterinary practitioners may need to upskill in foetal pathology and consider providing this new service for their clients where this is legally allowed. Veterinary diagnosticians can review the investigative protocols they currently adopt to see where they can improve their investigative/necropsy/sampling/testing SOPs. Additionally, the educational role of veterinary diagnosticians to both veterinary practitioners and farmers can contribute substantially to improved field investigations. At an international level, the current lack of homogeneity in investigative approaches highlights the need for procedural standardisation [9]. While the focus of this review is on dairy foeto-perinatal loss, the material is equally applicable to beef foeto-perinatal loss [10]. Additionally, frequent reference is made to aspects of investigation of human perinatal mortality, where relevant.

## Case definitions

Both abortion and stillbirth are variously defined in the literature and in common usage by scientists, veterinary practitioners and by farmers. While viability-based definitions are occasionally used, e.g. nonviable foetuses are considered aborted irrespective of gestation length (foetal mortality), gestation-based definitions are recommended [11]. These definitions differ both inter- and intra-nationally.

### ‘Observable abortion’

By scientific consensus a bovine abortion has been defined as the expulsion of a foetus between the completion of differentiation (day 42) and the limit of foetal independent viability (day 260), [11]. Day 42 also represented the typical timing of manual pregnancy detection when this definition was formulated, whereas now with early (~ 30 days) and repeat (~ 60 days) ultrasonographic pregnancy detection the late embryonic and early foetal loss (LEEFL) periods are often combined. There are numerous foetal loss definitions used internationally. For example, in France a hybrid definition of abortion is used encompassing both abortion and perinatal mortality; expulsion of a foetus from 42 days of pregnancy onwards either stillborn or dying within 48 h of birth [12]. Whilst including early (42–120 d) foetal mortality is scientifically valid, in practice animals which abort this early generally return to oestrus without a foetus being expelled (resorbed) or observed (too small). Such returns-to-service have been advocated as an indicator of first and second trimester abortion (inferred abortion) in syndromic surveillance systems [13]. The low observation rate of first trimester abortions was confirmed in a US study where only 9% of abortions < 125 days were observed, while 41% of abortions between day 125 and 245 were observed [14].

Thus, from a veterinary practitioner and their clients’ perspectives, abortion may be defined as the expulsion of a non-independently-viable foetus before full-term that is likely to be observed (and possibly submitted to a laboratory for examination) by a farmer, i.e. an ‘observable abortion’. Irish regional veterinary laboratory data indicate that the period of risk for an observable abortion is from 120 to 260 days of gestation; over 95% of aborted foetuses submitted to a veterinary laboratory over a 25 year period were from the fourth month of pregnancy on (> 120 days), (Table 1) [15]. The foetus at 120 days is the size of a small cat with a crown rump length (CRL) of 20-30 cm and weighs ~ 1 kg. These data support the findings [16] that the majority of laboratory-investigated abortion cases are from the last trimester, while the majority of non-submitted abortions are from the first two trimesters; this leads to under-estimation of real abortion rates.

**Table 1:** Gestational age of veterinary laboratory-investigated aborted foetuses

Trimester	Month of gestation	Foetuses (No.)	%
First (early)	2	73	0.7
	3	298	2.9
Second (mid)	4	841	8.2
	5	1393	13.7
	6	2069	20.3
Third (late)	7	2858	28.0
	8	2661	26.1

Source: Cork Regional Veterinary Laboratory, DAFM, 10,193 foetuses over 25 years (1980–2003)

### ‘Peristillbirth’

Perinatal mortality may be defined as death of the foetus or perinate before, during or within 48 h of calving at full term (> 260 days); it includes both stillbirth and early neonatal mortality [17]. While ‘stillbirth’ is commonly used as a synonym for perinatal mortality, particularly by farmers and their veterinary practitioners, it is better defined as the death of a foetus before or during calving at full term. A novel portmanteau ‘peristillbirth’ (incorporating both stillbirth and perinatal mortality) may avoid this terminological imprecision. These gestational and perinate age thresholds are arbitrary and vary both intra- and inter-nationally.

In cases of indeterminate gestational age (e.g. unrecorded natural services), surrogate objective variables may be used as with babies, most commonly, birth weight and CRL. However, both have limitations. In cattle there are inadequate breed and plurality-adjusted gestational age body weight norm reference data to assess when a birth weight might be more indicative of an abortion or a peristillbirth. In order to address this issue the data in Table 2 have been compiled from research herds with excellent records to provide guidance on different full-term birth weights for various common breed and crossbreed, and plurality groups. The same principle is used by the WHO to define human perinatal mortality cases [18]. From these data the following breed and plurality-adjusted body weight norms for term calves are indicated: ‘Jerseys’ (Jersey or Jersey x dams mated to Jersey or Jersey x sires); > 15 kg for singletons or twins, ‘Jersey crossbreds’ [Jersey or Jersey x dams or sires mated to non-Jersey or Jersey x dams or sires, respectively, (i.e. only the dam or the sire is Jersey or Jersey x, not both)]; > 20 kg for singletons or twins, ‘Non-Jersey’ [other dairy breed dams (non-Jersey or Jersey x) mated to other dairy sires (non-Jersey or Jersey x) or beef sires]; > 25 and > 20 kg for singletons and twins, respectively.

**Table 2:** Birth weights (kg) (no. records, mean, mean + 2SD, range) and gestation length (Ges, days) of fullterm (260–300 days), singleton (*n* = 10,422) and twin (*n* = 615) calves of different breeds and crossbreeds

Dam genotype	Sire genotype	Singletons					Twins				
		No. records	Birth wt. mean	Birth wt. mean + 2SD	Birth wt. range	Ges. days	No. records	Birth wt. mean	Birth wt. mean + 2SD	Birth wt. range	Ges. days
Jersey, JerseyX	Jersey, JerseyX	200	26.21	16.54–35.88	14–45	281.15	6	21.83	10.92- 32.74	15–30	284.66
Jersey, JerseyX or dairy	Jersey, JerseyX or dairy	919	32.63	20.31–44.95	14–51	280.34	32	26.63	22.04- 31.22	15–34	277.87
Dairy (excl. Je, JeX)^a^	Dairy (excl. Je, JeX)	7960	41.84	29.61–54.07	11 to 76	281.62	496	34.14	23.18–45.1	18–55	277.02
Dairy (excl. Je, JeX)	Beef^b^	1343	42.82	30.86–54.78	18–63	283.6	81	35.07	22.45–47.69	21–51	278.86

^a^Dairy (excl. Je, Jex) = Holstein-Friesian, Friesian, Friesianx, Ayrshire, Norwegian Red, Norwegian Redx, Swedish Red,

Swedish Redx, Montbeliarde, Montbeliardex, Normande, Normandex

^b^Beef = Aberdeen Angus, Belgian Blue, Blonde D’Aquitaine, Charolais, Hereford, Limousin, Simmental

Source: Teagasc dairy research herds

## Reporting of foetal/perinatal mortality

Before addressing the titular topic; investigation, the issue of reporting needs to be addressed as it is equally if not more important.

### Under-reporting

Under-reporting is a major issue with bovine foetal/perinatal mortality internationally (the ‘untagged calf loss phenomenon’). To some risk-averse farmers the loss of a single valuable pregnancy may trigger a veterinary investigation (threshold for investigation) while for the majority of farmers multiple losses need to occur before this threshold is exceeded. A few abortions in large herds are ‘expected’, i.e. abortion is considered a ‘normal’ event as long as it is sporadic. In a French study only 20% of beef farmers who had an abortion or stillbirth (0.8% abortion rate) reported it, indicating the low sensitivity of event-driven or passive abortion surveillance [19]. A foetal mortality rate of > 5% is the investigation threshold used by the majority of veterinary practitioners in the EU [20]. However, a cluster of cases is a more important ‘tipping point’ for investigation in seasonal calving herds, than percentage of pregnancies aborted. Scan statistic probabilities can be used to determine what constitutes a significant clustering of abortions [1]. Given the diagnostic gap between 42 and 120 days of pregnancy when aborted foetuses are generally not observed or reported/submitted for examination, return-to-service has been proposed as a proxy syndromic surveillance indicator to monitor losses in this period [13]. Proximity to the laboratory, number of losses, ability to identify the aborted cow, fear of a contagious abortifacient (e.g. *Brucella abortus*), ‘farm blindness’ [21] and lack of understanding of the costs and potential consequences of abortion influence the likelihood of sample submission. In addition, there is often a disconnect between the priority placed on reporting of abortion by governmental risk managers and veterinary practitioners and farmers [12].

The herd-level occurrence of abortions/stillbirths follows a right-skewed distribution with most herds having no or low losses but a minority of herds have high losses. The threshold at which loss prevalence becomes a ‘problem/cluster/storm’, and hence is investigated, is usually defined by the herdowner. Norm-referenced thresholds are cohort-based which may reflect the national recorded abortion or stillbirth rate. Criteria-referenced thresholds are based on metrics other than those based on cohorts, e.g. a theoretical or empirical threshold. While the latter has traditionally been 2% for abortions, this may only be appropriate for observable abortions (> 120 days) (in temperate regions) but may underestimate ‘normal’ all-cause, first through third trimester abortion rates.

## Under- and over-triage

The approach to a sporadic abortion/stillbirth or an autolysed foetus in a herd without a history of foetal loss problems will differ from that in a herd with a series of cases or a fresh case. This is referred to as under-triage, whereby such cases are not investigated as thoroughly as fresh cases or those from multiple mortality herds. However, over-triage may be warranted even for sporadic cases on farms a long distance from the local laboratory as resubmission of material is less likely. One cannot predict that the index abortion case is not the first of many in a herd outbreak (all abortion ‘storms’ start with a single case). Studies have shown that only approximately 20 to 30% of abortions are observed [22] suggesting that in many cases the first case presented (index case) is not in fact the first case that occurred, merely the first that was observed.

## Veterinary practitioner-led abortion/stillbirth investigation

When approached by a client with an aborted cow or a stillbirth problem (as defined above) the following standard operating procedure (SOP) is recommended.

## SOP for abortion/stillbirth investigation

This involves three steps; collect a history, examine pregnant animals and the aborted dam and examine the foetus/placenta. This procedure uses multiple diagnostic tools to make a clinico-pathological diagnosis [23, 24].

### Collect a history

The first step is to establish the nature and extent of the problem by investigating possible risk factors associated with the loss/es. Standard questions include how many cows have aborted/had stillbirths, how many cows are pregnant (denominator-based diagnosis), is the dam ill, are most losses in heifers, and recent husbandry changes. A pro forma questionnaire is used by veterinary laboratory staff to collect the history prior to the postmortem examination; practitioners might consider using such a format for problem herds – exemplars for abortions and peristillbirths are provided in Additional files 1 and 2. In addition to collecting information on the individual abortion/stillbirth, the herd health history is important. For example, details of most recent vaccinations against foetopathogens, recent cattle purchases and bulk milk test results.

### Examine/sample the pregnant and aborted/stillbirth animals

#### The pregnant cohort

Examining the pregnant animals and their environment allows the practitioner to assess their general health, body condition score (BCS) and feeding management. Cohort sampling of dams (*n* = 5–10) in the affected group can be useful in determining differences in exposure (presence/absence, prevalence) between affected and unaffected dams (case-control); but vaccinal status needs to be known.

#### The dam

Clinical examination of the dam may reveal pyrexia, diarrhoea, respiratory signs, etc. A faecal sample may be useful where salmonellosis is suspected. Single blood samples from aborted cows are the most common samples collected to investigate bovine abortion (sero-diagnosis). These can have moderate utility as proxy samples for foetal material. For example, a single blood sample from the non-vaccinated dam of an aborted or stillborn foetus can be up to 85% accurate in predicting a foetal culture-positive result for *Salmonella* Dublin, [25]. However, the primary value of maternal serum is as an exclusionary test for maternal antibody, i.e. a negative result rules out some causes, e.g. *Neospora caninum*.

Paired sera (more than 2 weeks apart) may detect rising titres (two to four-fold) for some abortifacients (e.g. *S.* Dublin SAT) but not for others (e.g. *Leptospira* spp., *Neospora*) due to the lag phase between infection and foetal mortality. In vaccinated herds natural infection can still be distinguished where DIVA vaccines are used (gE-deleted BoHV-I) and where titres are much higher than those expected from vaccination (e.g. *Leptospira*) this suggests current, active infection.

### Examine the foetus and placenta

#### Abortion/stillbirth foetopsy SOP

As with a clinical examination on a live animal a systematic approach means that important findings are not missed due to focusing on the obvious lesion/s; this involves three steps - external and internal examination and sampling the carcass. While for the aborted foetus few lesions are visible grossly, in the stillborn foetus lesions indicative of periparturient causes-of-death are more likely to be detected.

### External examination

The external examination will confirm the animal identification (assuming it is tagged), foetal preservation, maturity, size for date/plurality, and detect defects, discharges and predation. The stage of development of the foetus may be estimated from its body weight or size (e.g. CRL), (Table 3). External developmental indicators (dentition, pilosity, skull doming) are less useful due to their gradual appearance and subjectivity in assessment. Gestational age may be estimated (within plus or minus approximately 2 weeks) from foetometric variables such as straight CRL (sCRL) using simple formulae, for example: gestational age (GA), (days) = 68 + 2.25 x sCRL (cm) or GA = (sCRL + 21) × 2.5 or GA = 53 + (2.3 x SCRL) or more complex multi-variable formulae [26].

**Table 3:** Guideline physical characteristics of foetuses in the last two trimesters of gestation

Month of gestation	Body weight (kg)	sCRL^a^ (cm)	Pilosity (trunk/abdomen)	Teeth eruption (incisors)
6	5	50	Extremities only	Not erupted
7	15	70	Light hair coat	Partially erupted
8	30	90	Full hair coat	Partially or fully erupted
9	35	95	Full hair coat	Fully erupted

^a^sCRL = straight crown-rump length

Source: Teagasc research, Irish dairy herds

### Internal examination

How the carcass is opened and how the contents are examined varies between pathologists. An illustrated guide to bovine foetopsy by the practitioner has recently been published which details a simple method which can be carried out in a practice setting with minimal specialised equipment [27]. It is recommended that once the carcass is opened samples for microbiological testing are collected first to avoid contamination during the internal organ examination.

### Sampling the carcass

**Fig. 1:**
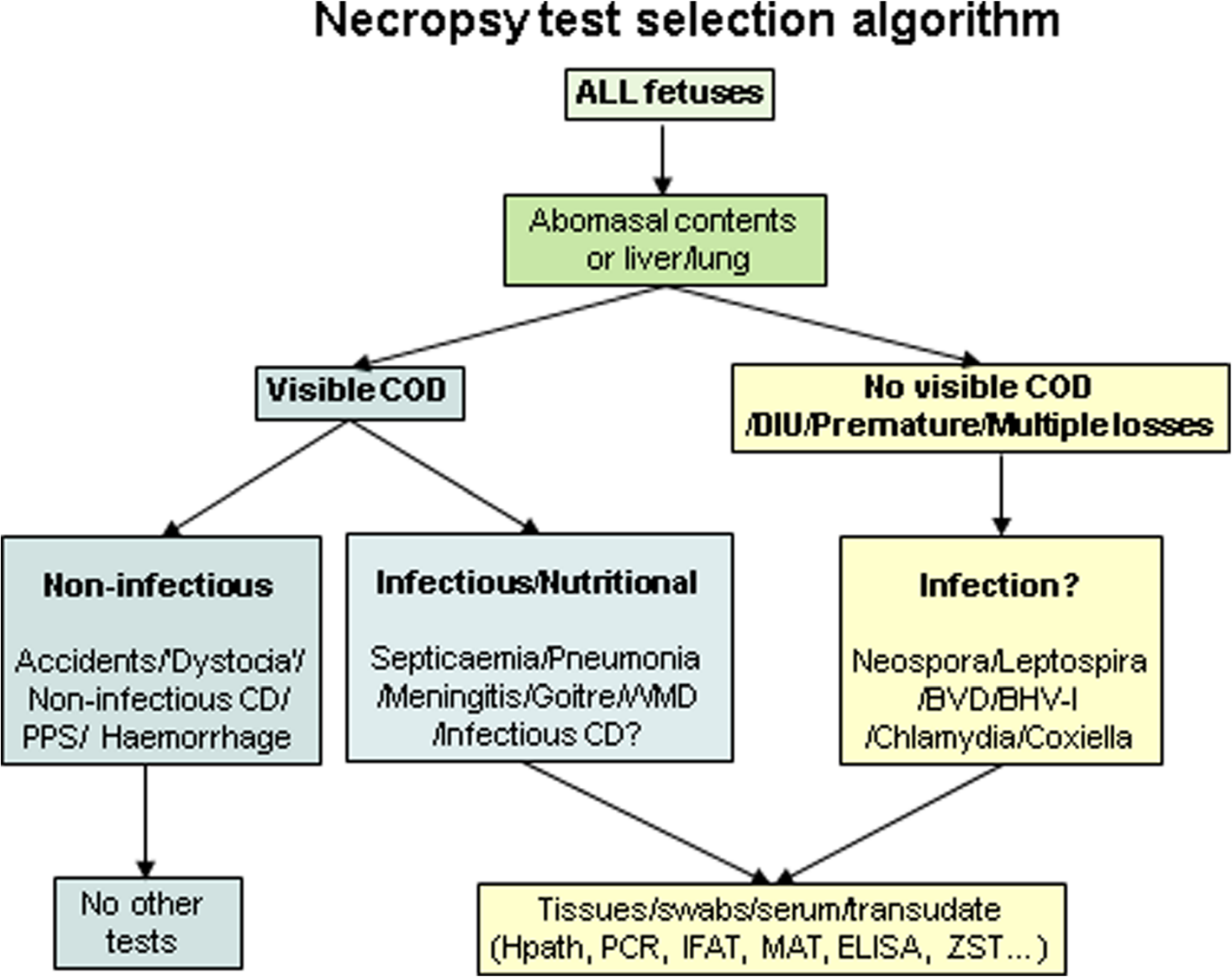
Practitioner-focused necropsy sample selection decision tree for aborted/stillborn foetuses (COD = cause-of-death, CD = congenital defect, DIU = dead in utero)

A sample selection algorithm for aborted/stillborn foetuses is outlined in Fig. 1. It is not possible to be prescriptive about test selection as laboratories differ in the test menu and tiers they offer. Variations between laboratories in sample and test selection both inter and intra-institutionally indicate the need for SOP harmonisation both nationally and internationally. Test selection will also be determined by the anamnesis, degree of carcass autolysis and costs. Rather than ‘necropsy by algorithm’ the decision-making of the practitioner or pathologist still determines whether and which samples to collect. Additionally, samples can be discarded if collected in the early stages of the necropsy where subsequent examination reveals the likely cause does not require laboratory testing.

#### Microbiology samples

Abomasal contents can be sampled aseptically by searing the abomasal serosa with a heated scalpel blade and aspirating a sample into a plain vaccutainer tube. In general, tissue samples are preferable to swabbed samples and surface swabbed samples are preferable to fluid swab samples. If an abomasal sample is unobtainable (due to scavenging or colostrum ingestion) or if septicaemia is suspected, lung, liver or brain samples are suitable alternatives, though not as sensitive [28]. Brain sampling is of particular value in severely scavenged and mummified foetuses. The presences of a pure growth of a pathogen with associated lesions in the absence of other causes of death are usually accepted as diagnostic criteria. However, recent research on variations in pathogen virulence (e.g. *Trueperella pyogenes*) suggest that in future it may be necessary to determine the presence of virulence factors also [29].

#### Serology samples

Serological sampling can be useful from the second trimester (> 120 days) when the foetus is immunocompetent as antibodies indicate foetal infection (but not necessarily foetopathy, e.g. *Neopsora* congenital infection), assuming placental competence. However, reliance on foetal serology alone may grossly underestimate foetal infection rates [30]. Recently, other biomarkers (acute phase proteins and non-pathogen specific immunoglobulins) have also been shown to be detectable in bovine foetal infection [31].

#### Trace element samples

Examination of the foetal thyroid gland for absolute goitre (thyroid enlarged relative to a criterion-referenced threshold thyroid weight, e.g. > 30 g) or relative goitre (thyroid enlarged relative to a criterion-referenced threshold thyroid g: kg ratio with body weight, e.g. > 0.80) and submission of a fresh (I_2_ content) and formalinised lobe (histopathology) will detect dietary iodine imbalance. Where selenium deficiency is suspected (possibly in conjunction with iodine deficiency) a sample of the foetal liver preferably [32, 33] or kidneys should be submitted. Given the associations between micronutrient imbalances and bovine abortion [34] and peristillbirth [35] examination of micronutrient status should be included in the diagnostic panel when investigating bovine foetopathy.

#### Histopathology samples

Samples for histopathology should include normal and abnormal tissue and should not be greater than 1 cm thick and 2 cm long as they need to fit into processing cassettes (3.5 × 2.5 × 0.5 cm); most samples collected by practitioners are too thick. Pathologists routinely sample lung, liver, thyroid, heart and brain. All samples can be submitted in a single pottle. As the brain is of particular value in the histopathological diagnosis of neosporosis, submission of the skull or entire brain is recommended as histopathologists vary in the sections they prefer to examine.

#### Standard abortion package

Practitioners will be guided by their local laboratory about which samples they prefer to use routinely for each test (‘standard abortion package’) and which tests are non-routine (e.g. PCR, histopathology). While for sporadic cases the basic sampling package may suffice (Table 4), in abortion storms, tiered sample escalation is advised as it provides the option of sampling, storing and testing as deemed necessary. Increasingly, genomic tests (e.g. dense SNPs) are being developed for developmental disorders which require specialist sampling, e.g. hair follicles, skin, muscle or liver.

**Table 4:** Standard and additional samples to collect from aborted and stillborn foetuses* for the investigation of infectious, nutritional and genetic causes of death

For investigation of	Standard samples	Ancillary samples	Comments
Failure of passive transfer of immunoglobulins	Perinate blood	NA**	Only test calves > 24 h old, e.g. ZSTT
Foetopathogenic bacteria and fungi (e.g. *Aspergillus* spp., *B. licheniformis, L. monocytogenes, T. pyogenes*, *S.* Dublin)	Foetal stomach contents (FSC), Placenta	Foetal lung, liver, gall bladder, kidney, brain, eyelid. Dam vaginal swab, placentome, blood.	Ancillary samples where FSC/placenta unavailable/contaminated.
*Neospora caninum*	Foetal brain, serum	Foetal heart. Placenta. Dam/cohort bloods	Fresh brain/placenta for PCR, fixed brain or heart/placenta for histopathology if PCR positive
*Leptospira Hardjo*	Foetal kidney, serum	Dam/cohort bloods	Foetal sample dependent upon laboratory tests
BVDv	Foetal ear, spleen, thymus, serum	Foetal kidney. Dam/cohort bloods	Foetal sample dependent upon laboratory tests
BHV-I	Foetal liver, serum.	Foetal kidney. Placenta. Dam/cohort bloods	Foetal PCR/histopathology preferred tests
Micronutrient deficiencies	Foetal thyroid, liver, heart, rib	Foetal kidney. Dam/cohort bloods	Thyroid for iodine assay/histopath; liver/kidney for selenium assay, heart for histopath; bone for manganese assay
Gross lesions (e.g. foetal pneumonia)	Affected foetal organ	As required	As appropriate (e.g. bacteriology, histopath)
Genetic congenital defect	Foetal muscle, skin	Dam hair follicles	Test for infectious teratogens also (e.g. BVDv, SBV)

*Standard and ancillary testing protocols are dependent upon local laboratory SOPs. Bacteriology/mycology (culture, stains, wet preparations) and serology are generally routine tests for sporadic cases while other tests (e.g. histopathology, PCR, FAT, IHC, micronutrient, DNA assay) can be added for multiple losses or at the discretion of the pathologist. Maternal vaccinal status affects use and choice of serology tests, ** NA - not applicable

#### Photo-documentation

Photo-documentation can be useful for inexperienced practitioners and for unusual lesions [36]. In future practitioners (and possibly farmers) may be able to use digital imaging technologies to enhance their PME yield, as currently occurs on North American feedlots (remote digital PME).

#### Sample submission

If samples are collected on a day when the laboratory is closed the samples should be stored for culture, serology and histopathology in a fridge (4 °C) and those for PCR in a freezer (− 20 °C). Practitioners need to be cognisant of postal regulations pertaining to packaging and labelling for biological substances. Completion of the receiving laboratory submission sheet and inclusion with the samples will facilitate timely processing of samples.

#### Examining the placenta

If the placenta has not yet been expelled at examination, a sample of the retained placentomes or a vaginal swab can be obtained. A normal placenta at term weighs approximately 5 kg, has 75–125 red cotyledons and has thin, translucent intercotyledonary tissue that sometimes contains adventitious placentation. Placentitis may manifest as discoloured, necrotic, exudative cotyledons and opaque, erythematous, thickened, oedematous intercotyledonary tissue. Ideally three abnormal cotyledons/inter-cotyledonary tissue samples should be submitted (preferably macroscopically abnormal and uncontaminated) for histopathological and microbiological examination (Gram smear, MZN, culture, PCR).

## Time-of-death

Before attributing a cause-of-death, the time-of-death (TOD) should be determined as the latter influences the diagnosis of the former. Time-of-death is classified as pre-, intra- and postpartum. Foetuses which die before birth will have varying degrees of autolysis; corneal opacity, excess body cavity sero-sanguinous transudate, decomposed organs (especially kidneys, liver, spleen and brain), haemoglobin imbibition, flaccid musculature, subserosal and subcutaneous serosanguinous oedema/emphysema and atelectatic lungs. Autolytic lesions develop within approximately 12 h of death in utero and become more advanced over time. Foetuses which were alive at the beginning of calving but die during birth or within an hour of calving will not have signs of autolysis and may have signs of breathing (partial lung inflation), bradytocia (localised peripheral oedema) or traumotocia (trauma lesions with haemorrhage). Perinates which die more than an hour after calving will not have signs of autolysis and will have signs of breathing (complete lung inflation, tracheal froth), and of umbilical cord rupture while alive [large (> 4 mm) diameter, pointed umbilical thrombi], possibly attempting to stand or standing (worn eponychia/foetal hoof ‘slippers’, especially hind) and colostrum consumption (colostrum in abomasum).

## Cause-of-death

Cause-of-death (COD) may be defined as ultimate/underlying (UCOD), (the first initiating event in the chain of events resulting in death; this often includes the clinical history, e.g. dystocia), intermediate and proximate/terminal (PCOD), (final event/mechanism causing death; often from the necropsy examination, e.g. fractured spine or asphyxia). The UCOD answers the question ‘*why* did the calf die?’, and the PCOD answers a subtly different question ‘*how* did the calf die? The fundamental question about the aetiology of perinatal mortality is ‘*why did the calf die?*’ i.e. what was the ultimate cause of death? For stillborn babies, the WHO recommends recording of the main single, underlying disease/condition causing death on the WHO Death Certificate [37]. In contrast, in veterinary studies the proximate COD from necropsy results is usually cited, as the submitted history is often incomplete. These COD concepts are important in identifying and mitigating underlying effects to prevent future deaths. Thus, shifting the focus from proximate to antecedent COD is recommended in veterinary studies. Determination of the COD is also fraught with the diagnostic challenge of trying to differentiate ‘death *with*’ from ‘death *from*’. Additionally, while most diagnoses are ‘diagnoses by presence’, ‘diagnosis by exclusion’/idiopathy is also employed where the former criteria cannot be satisfied, e.g. non-parturient trauma.

Common COD in peristillbirths based on an international Delphi study [9] are listed in Tables 5 and 6; other uncommon causes also occur. For babies, an extensive list of COD has been codified by the WHO in ICD-PM [37]. These can be used to calculate cause-specific mortality rates and the attributable fraction of peristillbirth due to each COD. The degree of confidence in diagnosing the COD can vary from definite (e.g., intralesional foetopathogen detected) to probable (foetopathogen detected and only gross lesions) and possible (foetopathogen detected but no lesions) depending on the extent of the investigation and the findings [38]. Additionally, current understanding on COD is constantly being informed by new research, for example, the detection of foetopathogens in the pregnant uterus of healthy cows in the absence of inflammation [39].

**Table 5:** Cause-of-death and diagnostic case definitions for non-infectious causes of bovine perinatal mortality

Cause-of-death	Sub-category	Case definition	Diagnostic criteria
**Accident**	Colostrum aspiration (iatrogenic)	Administration of colostrum by oro-oesophageal feeder or bottle into the trachea with subsequent calf clinical signs (e.g. bawling, dyspnoea, weakness, depression, recumbence).	History and presence of colostrum in the trachea, bronchial tree and lungs, pulmonary oedema/congestion/consolidation/pneumonia and foreign basophilic deposit in airways and alveoli
	Non-parturient trauma	Trauma independent of calving such as stood on or laid on by cow or attacked by cow (assault injury) or otherwise fatally injured, e.g. by automatic scraper in cubicle house, other machinery.	History and/or fatal traumatic lesions (e.g. antemortem fractured ribs and/or legs, hepatic rupture) usually in the absence of subcutaneous bruising.
	Oesophageal rupture (iatrogenic)	Rupture of oesophagus while administering colostrum using an oro-oesophageal feeder with subsequent calf clinical signs (e.g. bawling, dyspnoea, weakness, depression, recumbence, cervical oedema).	History and traumatic tear in oesophagus with discharge of colostrum and cellulitis if in cervical region resulting in a swollen neck.
**Co-mortality**		Presence of more than one cause-of-death	Listed for each cause-of-death
**Congenital defect**	Lethal congenital defect	Defect present at birth incompatible with life. Where the cause of the defect/s is diagnosed, e.g. BVDv, both an ultimate and proximate COD can be reported.	Most diagnosed defects are grossly visible structural defects. Examples include hydranencephaly, hydrocephalus, schistosomus reflexus and multiple defects. Some defects are economically lethal – the calf may survive following remediation but this is economically prohibitive hence euthanasia follows.
	Economically-lethal congenital defect	Grossly visible structural defect incompatible with independent life and with economic viability of the calf (e.g. surgery may be possible but cost-prohibitive and poor prognosis) necessitating euthanasia	Examples include intestinal atresia, vestigial limbs, palatoschisis and arthrogryposis.
**Dystocia**^a^	Bradytocia	Prolonged stage one or two of calving	History of prolonged stage one (e.g. milk fever, ‘slow calving syndrome’, disturbance during calving, uterine torsion) and/or prolonged stage two (e.g. foetal oversize) with moderate/severe peripheral subcutaneous antemortem oedema (e.g. lower legs, tongue, submandibular, head, neck)
	Dystoxia	Dystocia with anoxia/asphyxia lesions	Moderate/severe calving assistance with atelectasis and moderate/severe meconium staining/aspiration (hair, trachea, lungs, abomasum), mucosal/serosal haemorrhages (e.g. trachea, heart, pleura, thymus, abomasum, adrenals, sclera, conjuctiva), organ congestion and thoracic/abdominal serous transudate.
	Maldistoxia	Maldisposition with anoxia/asphyxia lesions	Malpresentation or malposition with moderate/severe meconium staining/aspiration (hair, trachea, lungs, abomasum), mucosal/serosal haemorrhages (e.g. trachea, heart, pleura, thymus, abomasum, adrenals, sclera, conjuctiva), organ congestion, thoracic/abdominal serous transudate and atelectasis.
	Traumotocia	Fatal trauma to the calf at assisted calving	Severe antemortem (haemorrhage at the site) acute lesions consistent with history of iatrogenic parturient trauma (e.g. fractured/dislocated spine, ribs, limbs, moderate/severe subcutaneous thoracic and lower limb haemorrhage/bruising, traumatic diaphragmatic hernia, hepatic rupture, moderate/severe haemothorax, haemoperitoneum, haemarthrosis, or polytrauma)
**Eutoxia**		Eutocia with anoxia/asphyxia lesions, e.g. umbilical cord accidents, placental insufficiency, placentitis, ‘non-clinical dystocia’.	History of no (or slight) calving assistance with some or all of the following: moderate/severe amniotic fluid or meconium staining/aspiration [e.g. lungs (multifocal keratinocytes, exfoliated epithelia, yellow/brown granular material, eosinophilic material in alveoli and bronchioles, incipient inflammatory reaction), hair, trachea, abomasum], mucosal/serosal petechial haemorrhages (e.g. trachea, heart, pleura, thymus, abomasum, adrenals, sclera, conjuctiva), organ congestion, thoracic/abdominal serous transudate and atelectasis (dark purple, moist, congested, heavy fluid-filled, round-bordered lungs; negative on floatation test)
**Haemorrhage/ anaemia**	Anaemia	Generalised pallor in the absence of visible haemorrhage	Diffuse severe pallor (e.g. conjunctiva, gingiva, skeletal muscles, thymus, trachea, liver, heart, lungs, brain, adrenals, kidneys)
	Omphallorhagia	Haemorrhage from the umbilical arteries	Severe, acute peri-umbilical haematoma or moderate/severe hemoperitoneum (up to > 1 l free blood and coagulum) with one or more unsealed umbilical arteries (internal omphallorhagia) and diffuse pallor with/without blood stained hair coat (external omphallorhagia)
**Hypothermia**		Cold-stress induced mortality	History of extreme cold weather stress (~ < 0 °C, high wind speed, rain/sleet/snow; wind chill), unobserved calving, not born into a straw bed (e.g. cubicle house, outdoors), not dried off (wet/faeces covered coat, abandoned by dam), no colostrum consumption (FPT), low rectal temperature (< 37 °C) and limb/ventral sternum lesions (yellow subcutaneous oedema and haemorrhage, esp. hind legs)
**Micronutrient disorder**	**Iodine/selenium imbalance**	Abnormal thyroid gland	Thyroid histopathological lesions: hyper- (current imbalance: columnar epithelium-lined microfollicles +/− epithelia invaginations) or hypoplasia (historic imbalance: moderately/markedly enlarged colloid-filled follicles lined by flat columnar epithelium; ‘colloid goitre’) and atelectasis with/without low thyroid iodine (< 1200 ppm DM), low tissue selenium content, absolute (thyroid > 30 g) or relative (thyroid/body weight ratio > 0.80) goitre
	**Selenium/Vitamin E deficiency**	Cardiomyopathy with low selenium/vitamin E status (white muscle disease)	Gross/histological lesions (cardiomyopathy - streaks of myocardial pallor with hyaline necrosis +/− calcification; Zenkers’ necrosis) plus low tissue (e.g. liver, kidney) or blood selenium and/or vitamin E concentrations
**Premature placental separation (PPS)**		Separation and expulsion of the placenta before/with the calf	History of placenta expelled before/with the fresh foetus and/or placenta attached to fresh carcass, Grossly the placenta may be normal. On histology there may be acute, multifocal haemorrhage into the interstitium of the cotyledonary villi.
**Prematurity or dysmaturity**		Calf born before physiological maturity	Premature characteristics [e.g. small size (IUGR), short light hair, partial incisor eruption, domed skull, respiratory distress syndrome (RDS)] in fullterm (dysmature) or pre-term [(premature; <mean-2SD gestation for single (< 270 days) or twin (< 265 days)] foetuses (e.g. sudden unexpected calving, poor udder swelling and pelvic ligament relaxation)
**Twin-to-twin transfusion**		Transfusion of blood from one twin to the other resulting in one anaemic and one congested twin with fatal sequelae	Foetal plurality with asymmetrical body weights if chronic (IUGR and normosomia) and asymmetrical carcass lividity (pallor and congestion)
**Unexplained**		Idiopathic stillbirth	None of the diagnostic criteria of causes-of-death apply; no visible/no significant lesions (NVL/NSL) or diagnosis not reached (DNR)

^a^Polypathia may also occur, e.g. bradytraumotocia, bradytoxocia, bradytraumotoxocia, traumotoxocia; where known, clinical history can be combined with pathology to give a clinicopathological case definition, e.g. bradytocia-maldisposition

**Table 6:** Cause-of-death and diagnostic case definitions for infectious causes of bovine perinatal mortality

Cause-of-death	Sub-category	Case definition	Diagnostic criteria
**Infection**	Foetopathogenic bacteria	Infection with *a significant bacterium: Bacillus licheniformis, Campylobacter foetus, Listeria monocytogenes, Salmonella* Dublin*, Trueperella pyogenes*, etc	Pure heavy growth in culture of pathogenic species from abomasal contents/foetal tissues or nearly pure/mixed growth with associated lesions consistent with foetal sepsis/placentitis (in particular for potential contaminants, e.g. *E. coli*). If isolated from placenta only, associated intra-lesional bacterial placentitis.
		*Chlamydophilia/Parachlamydiacae*	Detection of organism in the placenta, foetal tissue or abomasal contents (e.g. MZN smear, PCR, IHC) confirmed by histopathology
		*Coxiella burnetii*	Detection of organism in the placenta, foetal tissue or abomasal contents (e.g. MZN smear, PCR, IHC) confirmed by histopathological lesions (e.g. necrotising placentitis); Detection of the organism in the absence of lesions indicates very acute recent infection.
		*Leptospira spp (*e.g. *Hardjo, Grippotyphosa, Australis)*	Detection of pathogenic antigen (e.g. PCR/FAT/IHC-positive) in foetal tissues (e.g. kidney, spleen) with accompanying lesions or detection of high foetal antibody titre indicating recent infection
	Foetopathogenic fungi	*Aspergillus app, zygomycetes, yeasts, etc*	Detection of fungal hyphea in abomasal contents/placenta (e.g. wet prep, culture) with associated gross/histopathology lesions (e.g. foetal dermatitis, IUGR, placentitis, hepatomegaly)
	Foetopathogenic parasite	*Neospora caninum*	Detection of histological lesions (characteristic neuropathology, myocardial necrosis, multifocal placentitis; cause) and the parasite antigen in foetal tissues (e.g. PCR; infection) +/− foetal antibodies (exposure)
	Foetopathogenic viruses	BoHV-I, 4	Detection of viral antigen in foetal tissues (e.g. liver, spleen, adrenal) +/− lesions, e.g. focal necrotising hepatitis, placentitis
		BVDv	Detection of viral antigen in foetal tissues (e.g. spleen, thymus, adrenal, ear) and gross/histopathology lesions
		SBV	Detection of two or more syndromic gross lesions (arthrogryposis, hydranencephaly, torticollis, scoliosis, kyphosis, brachygnathia inferior) and foetal antigen (e.g. PCR).
	Infectious lesions	Compelling lesions indicative of infection	Gross/histological lesions consistent with exposure/response to infection e.g. pericarditis, meningoencephalitis, enteritis omphalo-peritonitis, pleuropneumonia, lymphadenomegaly, systemic sepsis (lesions in at least two organs) and placentitis.

The relative proportions of various COD can be influenced by the surveillance model used to collect data; an active surveillance model gives an accurate picture of whole-herd mortality while scanning/passive surveillance may give a biased picture of preselected cases and an underestimation of low incidence cases.

Diagnosis rates in aborted and stillborn foetuses are generally between 20 to 50% [5] and 30 to 75%, [6], respectively. The more carcasses and the fresher the carcasses that are examined the higher the herd diagnosis rate. In aborted foetuses the COD are often attributed to events which occurred days or weeks before foetal death so few COD lesions will be grossly visible; microbiological sampling is more important. However, in stillborn calves the COD is more likely to be periparturient [40] rather than infection-related [41] though pre-existing congenital defects [42] and precalving nutrition [43] can also contribute to perinatal mortality. Hence gross examination can often reveal more than laboratory testing. Foetopathogenic infections (Table 7) and dystocia (abnormal calving), (Table 8) continue to be the most important diagnosable causes of bovine abortion and stillbirth, respectively. The predominant infectious agents causing abortion vary by country, with *Neospora caninum* frequently cited as the most common pathogen internationally [45]. The predominant types of dystocia causing stillbirth are foetal maldisposition (of presentation or posture), bradytocia (prolonged stage one or two of calving), and traumotocia (calving-associated foetal trauma). Foetal mortality in many cases of dystocia is caused by perinatal asphyxia. Co-mortality (polypathia) is not uncommon in stillbirths and should be considered; in some cases the main and other causes may be distinguishable and in other cases not. For example, immunocompromise in goitrous perinates leading to increased susceptibility to septicaemia [46]. The WHO recommends recording of other significant conditions contributing to perinatal mortality [37].

**Table 7:** Detection of foetopathogens in Irish aborted and stillborn foetuses and placentae

Organism	%
*Trueperella pyogenes*	6.8
*Bacillius licheniformis*	5.3
*Salmonella* Dublin	4.2
*Neospora caninum*	4.0^a^
*Listeria monocytogenes*	2.2
*Aspergillus* spp	0.6
Secondary bacterial and fungal spp.	7.4

^a^not all foetuses are tested for *Leptospira hardjo* (foetal ELISA) and *Neospora caninum* (foetal ELISA +/− histology), hence no figures are recorded for the former and those for the latter pathogen do not represent the full sampling frame (*n* = 1970 cases)

Source**:** Sanchez-Miguel, (2019), [44]

**Table 8:** Causes of parturient mortality (%) in dairy calves by calving assistance category

Cause of calf death (%)	Unobserved calving	Observed, no calving assistance	Easy calving assistance	Moderate calving difficulty	Severe calving difficulty
Maldisposition	0	2	18	29	28
Bradytocia	21	14	30	26	24
Congenital defects	8	8	4	9	18
Eutoxia	23	33	21	0	0
Infections	8	8	2	7	7
Other	29	44	22	27	16
No significant findings	11	6	3	2	7

Source: Teagasc research, Irish dairy herds

## Diagnosis not reached (DNR)

The common reasons for DNR are insufficient, poor quality or incorrect sample submission (especially non-submission of the placenta), diagnoses not attainable from PME, poor PME technique, and inadequate or unavailable laboratory tests. In recording DNR data it is useful to append the possible reason for the DNR to improve investigative yields in future studies. In the case of aborted foetuses and ‘unexplained stillbirths’ non-infectious and non-dystocial causes, respectively, are likely to be the major reasons for DNR. For example, recent studies showed a significantly higher abortion rate in cows fed diets with high mycotoxin concentrations [47] and a chromosomal deletion has been shown to be significantly associated with increased stillbirth in cattle [48]; these causes are not detectable in diagnostic (as opposed to research) veterinary laboratories. While DNR may appear to a client or a veterinary practitioner as a failure of investigation, it can be viewed as a successful rule-out of common causes of foetopathy.

## Conclusions

From this thematic review it may be concluded that we can improve and standardise our case definitions of bovine foetopathy, we can conduct better case investigations by adopting pro forma to collect information on the anamnesis, pregnant cohort, affected dam and her foetus/es, we can increase our necropsy yield by performing a standardised external and internal examination and sampling protocol while being cognisant of the need to establish the time-of-death relative to abortion or calving and the primacy of determining the ultimate, as opposed to the proximate cause of death, diagnoses not reached/reachable, notwithstanding.

## Supplementary information

**Additional file 1.** Peristillbirth questionnaire.

**Additional file 2.** Abortion questionnaire.

## Data Availability

All data generated or analysed during this study are included in this published article.

## Glossary

Bradytocia: prolonged stage one or stage two of calving
Co-mortality: more than one cause of mortality in the same calf
Eutocia: normal calving
Dystocia: abnormal, but not necessarily traumatic/difficult, calving
Foetal maldisposition: foetal malpresentation, malposture or malposition
Observable abortion: expulsion of a foetus which is likely to be observed, and hence submitted to a veterinary diagnostic laboratory; between day 120 and the limit of independent foetal viability (< 260 days)
Perinatal asphyxia: a combination of periparturient hypoxia, hypercapnia and metabolic and respiratory acidosis induced by foetal hypoperfusion/ischaemia (Greek; asphyxia = absence of pulse); synonym anoxia
Perinatal mortality: death of the foetus or perinate before, during or within 48 h of calving at full term (> 260 days); it includes both stillbirth (death of a foetus before or during calving at full term) and early neonatal mortality. Peristillbirth incorporates both stillbirth and perinatal mortality
Proximate/terminal cause-of-death: final event/mechanism causing death. This is often diagnosed from the necropsy examination based on pathological lesions, e.g. fractured spine.
Traumotocia: trauma of the foetus or dam during calving
Ultimate/underlying cause-of-death: the first initiating event in the chain of events resulting in death. Diagnosis often includes the clinical history and the necropsy examination, i.e. a clinico-pathological diagnosis, e.g. dystocia.
